# Multi-Modal Ultrasound-Based Prognostic Model for Diffuse Large B-Cell Lymphoma with Predominantly Superficial Lymph Node Involvement

**DOI:** 10.3390/diagnostics16152311

**Published:** 2026-07-23

**Authors:** Jingzhe Wang, Jie Mu, Yichen Yang, Xiangrui Meng, Yuting Song

**Affiliations:** 1 Department of Diagnostic and Therapeutic Ultrasonography, Tianjin Medical University Cancer Institute and Hospital, Tianjin 300060, China; mujie@tjmuch.com (J.M.); songyuting618@hotmail.com (Y.S.); 2 Tianjin Medical University, Tianjin 300060, China; yang_yichen@tmu.edu.cn (Y.Y.); xiangruim1984@163.com (X.M.); 3 Key Laboratory of Cancer Prevention and Therapy, Tianjin 300060, China; 4 Tianjin’s Clinical Research Center for Cancer, Tianjin 300060, China; 5 National Clinical Research Center for Cancer, Tianjin 300060, China; 6 Tianjin Cancer Institute, Tianjin Medical University Cancer Institute and Hospital, Tianjin 300060, China; 7 Department of Lymphoma, Tianjin Medical University Cancer Institute and Hospital, Tianjin 300060, China

**Keywords:** deep learning, diffuse large B-cell lymphoma, multi-modal ultrasound, nomogram, prognostic model, progression-free survival, radiomics, superficial lymph node

## Abstract

**Objective**: This study aimed to develop a multi-modal ultrasound-based model integrating radiomics, deep learning, and clinical features to predict progression-free survival (PFS) in diffuse large B-cell lymphoma (DLBCL) with superficial lymph node involvement. **Methods**: A total of 281 DLBCL patients with superficial lymph node involvement treated with standard regimens were retrospectively enrolled and assigned to training and test sets at a 7:3 ratio. Ultrasound radiomic features were extracted by PyRadiomics, and deep learning features were derived from DenseNet121 with transfer learning. After Pearson’s correlation filtering and least absolute shrinkage and selection operator (LASSO)-Cox regression for feature selection, five prognostic models were compared using C-index, time-dependent Area Under the Curve (AUC), risk stratification and decision curve analysis. **Results**: The combined model achieved the highest C-index of 0.811 in the test set, with 1-/2-/3-year PFS AUCs of 0.826, 0.812, and 0.810, respectively. Risk stratification showed significantly poorer PFS in the high-risk group (*p* < 0.01). A visualized nomogram was further developed as a preliminary reference for individualized prediction. **Conclusions**: This multi-modal combined predictive model and corresponding nomogram based on superficial lymph node features showed promising potential for practical and intuitive prognostic assessment and risk stratification in DLBCL.

## 1. Introduction

Diffuse large B-cell lymphoma (DLBCL) is the most common subtype of non-Hodgkin lymphoma (NHL), accounting for approximately 30% to 40% of all lymphoma cases [1]. Although the standard treatment with rituximab combined with cyclophosphamide, doxorubicin, vincristine and prednisone (the R-CHOP regimen) has significantly improved the prognosis of patients, 30% to 40% of patients still experience disease progression or relapse and fail to be cured, eventually succumbing to disease recurrence [2]. Therefore, identifying prognostic factors of DLBCL and establishing an effective prognostic model to screen high-risk DLBCL patients at the initial treatment who may develop recurrence and progression following standard therapy, thereby guiding the formulation of novel individualized treatment regimens, is of great significance in the current diagnosis and management.

At present, classic prognostic scoring systems such as the International Prognostic Index (IPI) and the National Comprehensive Cancer Network-International Prognostic Index (NCCN-IPI) have been widely used in the clinical management of DLBCL [3]. In addition, a number of studies have successively developed various prognostic models based on the clinic relevant data [4,5,6,7,8]. However, these models mostly rely on routine clinical data or require invasive and costly examinations, such as positron emission tomography (PET). Some studies failed to capture the core features, including tumor morphological heterogeneity and biological behavior, with limited predictive efficacy (the C-index is mostly between 0.65 and 0.72), which failed to meet the requirements for precise prognostic assessment. Carreras J. [9] showed that histopathological images could predict 2-year mortality, and Han et al. [10] integrated genomic features with clinical indicators to improve IPI-based stratification. However, the former requires expensively invasive biopsy and is not suitable for repeated monitoring, while the latter relies on genomic profiling, which remains costly and not universally available.

Imaging technology plays a pivotal clinical role in the diagnosis, staging and treatment monitoring of DLBCL. PET and magnetic resonance imaging (MRI) are not feasible for initial examination or treatment monitoring in some patients due to contraindications and high examination costs. Ultrasound is routinely used for initial lesion localization and preliminary assessment owing to its advantages of non-invasiveness, convenience, low cost and absence of radiation. Meanwhile, with the advances in ultrasound imaging quality and physician diagnostic proficiency in recent years, more DLBCL patients choose to rely on ultrasound for disease progression assessment and prediction. Nevertheless, conventional ultrasound largely depends on the subjective qualitative judgment of physicians, such as lesion boundary, shape, striated/reticular echogenicity, elastography score, with insufficient mining of multi-modal image information, making it difficult to fully reflect tumor biological characteristics. In recent years, as an emerging quantitative image analysis technology, radiomics can extract a large number of subvisual subtle features from medical images, quantify tumor morphological heterogeneity and achieve accurate mapping of biological behavior, showing promising potential in prognostic prediction for various malignancies [11,12,13]. Compared with handcrafted features, deep learning technology has further expanded the depth of image feature mining and demonstrated strong capabilities in detection, classification and prediction in medical image processing and analysis [12,13,14,15]. The in-depth mining of imaging features of cervical, axillary and inguinal superficial lymph nodes in DLBCL based on artificial intelligence (AI) technology is expected to break through the limitations of traditional qualitative diagnosis. Recent advances in deep learning have enabled robust detection and segmentation of superficial lymph node metastases using multi-modal ultrasound images [16]. These findings support the feasibility of applying similar techniques for prognostic prediction in DLBCL.

Several studies have confirmed that combined models integrating radiomics or deep learning with clinical indicators have significantly better prognostic predictive efficiency for DLBCL than a single model. For example, the combined model constructed based on metabolic imaging information extracted from PET images has been proven to have higher clinical value in the prognostic assessment of DLBCL patients [5,6,8]. MRI-based radiomic models integrated with clinical factors also exhibit excellent prognostic performance in patients with primary central nervous system DLBCL, performing significantly better than clinical-only models [17,18]. However, most of the existing imaging-based prognostic studies on DLBCL focus on modalities such as PET-CT and MRI, which are costly and associated with radiation exposure while yielding poor visualization of the fine structural details of superficial lymph nodes. However, ultrasound-based studies on DLBCL are primarily limited to diagnostic analysis of conventional ultrasound features without involving artificial intelligence (AI) technology [19,20,21]. There are few studies on constructing multi-feature combined prognostic models based on ultrasound and individualized prognostic assessment tools. Recently, Yu et al. demonstrated the feasibility of ultrasound-based radiomics and deep learning for overall survival prediction using a ResNet50 architecture [22]. However, their model did not simultaneously integrate multiple ultrasound modalities (e.g., elastography and superb microvascular imaging) into a unified input, potentially missing complementary information.

To address this gap, this study focused on the representative cervical, axillary and inguinal superficial lymph node lesion in DLBCL with superficial lymph node involvement, and constructed a progression-free survival (PFS) prediction model and corresponding nomogram by integrating their multi-modal ultrasound radiomic features, deep learning features and clinical indicators. Through rigorous feature screening and performance evaluation, we developed a pre-treatment, multi-modal ultrasound-based combined prognostic model for PFS in DLBCL patients that combines four ultrasound models using DenseNet121 with the goal of enabling risk stratification and individualized prognostic assessment through a preliminary nomogram.

## 2. Materials and Methods

### 2.1. Study Subject

DLBCL patients diagnosed at our hospital between March 2021 and December 2022 were retrospectively enrolled. Ultrasound images and clinical medical record data for this retrospective study were collected. This study was conducted in accordance with the Declaration of Helsinki, the protocol was approved by the Ethics Committee of Tianjin Medical University Cancer Institute and Hospital (bc20250203) on 13 January 2025, and the requirement for written informed consent from patients was waived.

Inclusion criteria: (1) pathologically confirmed as DLBCL in accordance with the criteria of the 5th Edition of the World Health Organization (WHO) Classification of Haematolymphoid Tumours [23]; (2) patients presented with superficial lymph node involvement (cervical, axillary, and inguinal regions) with or without deep nodal/extranodal involvement as the initial and predominant sites of involvement; (3) newly diagnosed patients without prior anti-tumor treatment such as chemotherapy, radiotherapy and targeted therapy; (4) complete ultrasound examination within 1 week before treatment with good image quality (no severe artifacts, with clear identification of lesion boundaries and internal structures); and (5) radical treatment with R-CHOP-like regimens.

Exclusion criteria: (1) patients with incomplete medical data, loss to follow-up or a history of previous cancer; (2) patients with contraindications to ultrasound examination or severe artifacts in images; (3) patients complicated with severe hepatic and renal insufficiency, immunodeficiency and other underlying diseases; and (4) patients with isolated involvement of deep lymph nodes (e.g., mediastinal and abdominal lymph nodes) or extranodal organs.

Clinical data of patients were recorded through outpatient and inpatient medical records, including gender, age, extranodal involvement, Ann Arbor stage, lactate dehydrogenase (LDH) level, ECOG performance status score, B symptoms, mass lesions and cell of origin. Ann Arbor stage and extranodal invasion were determined by physical examination and enhanced computed tomography (CT), and bone marrow aspiration and biopsy were performed for patients with clear indications. PET/CT results were excluded to reduce selection bias and the invasiveness of the model. Follow-up data were obtained through outpatient reexamination, electronic medical records and telephone interviews. Progression-free survival (PFS) was selected as the prognostic indicator for DLBCL patients. PFS was defined as the time interval from the date of diagnosis to the first recurrence, progression, death from any cause or the last follow-up. The survival status and disease progression time of patients were recorded during the follow-up period. The median follow-up time for the entire cohort was 27 months (interquartile range: 10–36.5 months). Overall, 158 of 281 patients (56.2%) were censored. The remaining 123 patients (43.8%) experienced a PFS event.

### 2.2. Ultrasound Examination Methods

Ultrasound examinations in this study were independently completed by two physicians with more than 5 years of experience in lymphoma ultrasound diagnosis. Standardized training was conducted for the operating physicians before the examination to ensure the consistency of examination procedures and parameter settings. Philips EPIQ 7 (Philips Healthcare, Bothell, WA, USA), Mindray Resona R9 (Mindray Medical International, Shenzhen, China), Canon Aplio 500 (Canon Medical Systems, Otawara, Japan), and GE LOGIQ E20 (GE Healthcare, Chicago, IL, USA) scanners equipped with high-frequency linear array transducers (frequency: 5~12 MHz) were used, and the central frequency of the transducers was fixed at 9 MHz to ensure the uniformity of image quality. Patients were placed in the supine position with a pillow under the shoulders and the head tilted back to fully expose the neck area, the axillary and the inguinal region clearly. Systematic scanning of superficial lymph nodes throughout the body was performed in the order of “neck → axilla → inguinal region”, combining continuous sliding scanning and fan-shaped scanning during the process to avoid missing lesions.

First, gray-scale two-dimensional ultrasound imaging (US2D) was performed: the instrument gain, depth and focal points were adjusted to place the lesion in the center of the image with clear display of internal structures, and the focal points were set at the central level of the lesion. Then, color Doppler flow imaging (CDFI) was conducted: the blood flow velocity range was set at 3~8 cm/s, the wall filter level was low, and the gain was adjusted to the point with just no color noise. Next, the superb micro-vascular imaging (SMI) mode was activated: high-sensitivity settings were adopted to clearly display the micro-vascular signals in the lesion. Finally, shear wave elastography (SE) was performed, keeping the transducer stable with appropriate pressure.

All ultrasound images were stored in JPG format. The US2D, CDFI, SMI and SE images of the target lesion in different sections were stored for each patient. If a patient had multiple lesions, the region of interest (ROI) was selected according to the principle of “priority to the largest diameter + priority to the most typical morphology”: lesions with a maximum diameter > 10 mm were preferred; if multiple lesions had similar maximum diameters, the lesion with malignant features such as blurred boundary, irregular shape, abnormal blood flow signal or high elastic hardness was selected as the target lesion; for a single lesion, it was directly used as the ROI for subsequent analysis. After the examination, the two physicians with 5 years of experience independently recorded the ultrasound features. In addition, all conventional US features showed excellent inter-observer consistency with ICC > 0.75. In case of diagnostic discrepancies, a third physician with more than 15 years of experience in lymphoma ultrasound diagnosis conducted an arbitration, and the arbitration result was used in the included data.

### 2.3. Data Partitioning

A total of 281 patients were enrolled in this retrospective study. The entire patient cohort was randomly stratified into a training set and an independent test set at a ratio of 7:3 to ensure the stability and generalization ability of model construction. All model development procedures, including feature screening, hyperparameter optimization, and model training, were performed exclusively on the training set to avoid data leakage and guarantee an unbiased evaluation. Specifically, for the radiomic signature, a five-fold cross-validation strategy combined with grid search was applied within the training cohort to identify the optimal hyperparameters for model construction. For the deep learning model, hyperparameters were determined with early stopping, as detailed in Section 2.6.4. After determining the optimal parameters, the final model was validated on the completely independent test set to objectively evaluate its predictive performance.

### 2.4. Tumor Segmentation

ROIs covering the entire tumor lesion were manually delineated on axial ultrasound images using ITK-SNAP software (version 3.8.0). The segmentation work was independently completed by two certified radiologists with more than 5 years of experience in oncologic ultrasound imaging, who were blinded to the clinical prognosis information. The vast majority of features demonstrated excellent reproducibility (ICC > 0.75) across all four modalities, with the percentage ranging from 82.99% to 98.14%. Any inter-observer discrepancies were resolved via consultation with a third senior radiologist with over 15 years of clinical experience for arbitration to obtain the final consensus on the ROI segmentation results.

**Figure 1:**
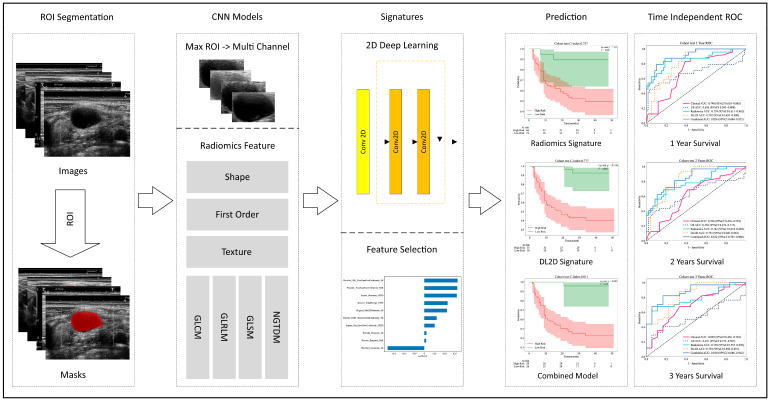
Schematic workflow of the ultrasound image analysis and survival prediction pipeline for DLBCL.

The subsequent sections detail the specific implementation of radiomic feature extraction, deep learning model construction, and model integration, as illustrated in the workflow below (Figure 1), which constitutes the core technical pipeline of this study for DLBCL prognostic prediction based on ultrasound images.

### 2.5. Radiomic Analysis

#### 2.5.1. Feature Extraction

For radiomic feature extraction, one axial slice per ultrasound modality was used, and 2D features were extracted from these slices. A total of 5152 radiomic features were extracted, with 1288 features derived from each of the four ultrasound modalities (US2D, CDFI, SE, and SMI). For each modality, features were computed independently from one predefined intra-tumoral compartment and subsequently concatenated using an early fusion strategy. All feature extraction was performed using the PyRadiomics platform (version 3.0.1), adhering strictly to the Image Biomarker Standardization Initiative (IBSI) guidelines to ensure the reproducibility and comparability of the results [24].

#### 2.5.2. Feature Selection and Radiomic Signature Construction

To mitigate the risk of overfitting caused by high-dimensional features, a rigorous three-step hierarchical feature selection procedure was performed. First, pairwise Pearson’s correlation coefficients were computed to remove redundant features, and only the feature with stronger univariate prognostic association (assessed by Cox proportional hazards regression) was retained for pairs with high correlation (|r| > 0.9). Second, univariate Cox regression was applied to filter features with significant prognostic value (*p* < 0.05). Finally, least absolute shrinkage and selection operator (LASSO)-Cox regression with 10-fold cross-validation was used to identify the optimal feature subset, where the optimal regularization parameter λ was determined by minimizing the partial likelihood deviance. Features with non-zero coefficients at the optimal λ were utilized to establish the final radiomic signature, and a multivariate Cox proportional hazard model was further constructed in the training cohort to calculate a personalized risk score for each patient.

### 2.6. Deep Learning Procedure

#### 2.6.1. Multichannel Input Construction

To integrate the complementary information of multiple ultrasound sequences, a multichannel input structure was constructed for deep learning model training. For each patient, the corresponding axial image slices of four modal sequences (US2D, CDFI, SE, SMI) were selected, and the tumor region was cropped with a fixed bounding box. These four cropped images were then stacked to form a single composite image with four channels. This approach allows the network to simultaneously learn and integrate features from all sequences within a unified representation. The input tensor can be formally defined as follows:

$$X\in R^{H\times W\times C}$$

where H and W represent the height and width of the cropped image patch, respectively, and C=4 corresponds to the number of input channels.

#### 2.6.2. Model Architecture and Transfer Learning

In this study, we built our model based on DenseNet121 and adopted a transfer learning strategy. To accommodate four-channel ultrasound inputs (vs. three-channel RGB for ImageNet pre-training), we adapted the first convolutional layer by changing its weight shape from [64, 3, 7, 7] to [64, 4, 7, 7]. The first three channels were initialized with ImageNet pre-trained weights and the fourth channel with zeros, thereby preserving pre-trained knowledge while learning the new modality. The adapted layer was fine-tuned along with the rest of the network. The original classification head was removed and replaced with a survival prediction head that outputs a continuous risk score (rather than binary probabilities), which was randomly initialized and trained from scratch. The entire network was then fine-tuned end-to-end on our dataset to learn task-specific features directly optimized for the prognostic task.

#### 2.6.3. Preprocessing and Data Augmentation

An ROI-centered image preprocessing flow was adopted. For each tumor-containing slice, a tight bounding box was generated according to the ROI mask, and a certain margin was expanded outward to include appropriate peritumoral tissue information. The image area within the bounding box was cropped to remove irrelevant background and retain key lesion information. All cropped images were uniformly resized to 224 × 224 pixels and normalized by Z-score standardization to meet the input requirements of the pre-trained CNN model.

During the model training phase, online data augmentation was used to improve the generalization ability of the model and reduce overfitting, including random horizontal flipping, vertical flipping, random cropping. During the validation and testing phases, only resizing and normalization operations were performed without any data augmentation to ensure the objectivity and repeatability of the evaluation.

#### 2.6.4. Model Training and Objective Function

The model was optimized using stochastic gradient descent (SGD), and the learning rate was dynamically adjusted using the cosine annealing strategy. The learning rate of the t-th iteration was calculated as follows:

$$\eta_{t}=\eta_{min}^{i}+\frac{1}{2}\left(\eta_{max}^{i}-\eta_{min}^{i}\right)\left(1+cos\left(\frac{T_{\mathrm{cur}}}{T_{i}}\pi \right)\right)$$

where $\eta_{min}^{i}=0$ and $\eta_{max}^{i}=0.01$ define the range of the learning rate for the i-th cycle, and $T_{i}=32$ sets the cycle length in epochs. This cyclical strategy helps the model converge efficiently and escape potentially sharp local minima.

To directly model the time-to-event nature of our survival data, the network was trained to minimize the negative log partial likelihood of the Cox proportional hazard model. For a given patient i with a predicted log-hazard $h_{\theta }\left(x_{i}\right)$ from the network, and a risk set $R_{i}$ comprising all patients still at risk at the event time $t_{i}$, the loss function is defined as follows:

$$L_{\mathrm{Cox}}=-\sum_{i:\delta_{i}=1}\left(h_{\theta }\left(x_{i}\right)-log\sum_{j\in R_{i}}e^{h_{\theta }\left(x_{j}\right)}\right)$$

where $\delta_{i}$ is the event indicator. Optimizing this function encourages the network to learn a hazard function that correctly orders patients by their risk of an event.

To prevent overfitting during end-to-end fine-tuning, we applied dropout (rate = 0.2) after the global average pooling layer and L2 weight decay (1 × 10^−4^). Early stopping was applied with a patience of 32 epochs, and restoration of the best-performing checkpoint was used to select the final model. The maximum number of epochs was set to 96, with a batch size of 32.

#### 2.6.5. DL2D Signature Generation

The continuous risk score predicted by the optimized DL2D model for each patient was extracted and defined as the DL2D signature. This signature represents a high-dimensional, image-derived prognostic biomarker for subsequent integration and analysis.

To improve the interpretability of the DL2D model and visualize its decision basis for prognosis, Gradient-weighted Class Activation Mapping (Grad-CAM) was applied to the final convolutional layer of the DenseNet121 network. This method generated activation maps that highlight the critical regions in ultrasound images used by the model to predict prognostic risk, thereby revealing the key imaging features for DLBCL PFS assessment.

### 2.7. Model Integration and Statistical Evaluation

#### 2.7.1. Clinical/US Signature Construction

Univariate Cox regression analysis was conducted on all clinical characteristics and conventional ultrasound indicators to screen for variables with significant prognostic value (*p* < 0.05). Subsequently, these significant variables were enrolled into a multivariate Cox regression model. The independent prognostic factors retained after adjustment were utilized to establish the clinical/ultrasound signature, and the corresponding risk score was calculated for each individual patient.

#### 2.7.2. Multi-Modal Combined Model

To evaluate the complementary value of the different data sources, an integrated model was developed. This model incorporated the radiomic signature, the DL2D signature, and the clinical signature as independent covariates within a unified multivariate Cox framework. This approach was designed to test whether the deep-learning-derived features provide prognostic information incremental to established clinical and handcrafted radiomic factors.

#### 2.7.3. Performance Assessment

The primary metric for model discrimination was the concordance index (C-index). For survival analysis and risk stratification, the optimal cut-off point for each continuous risk score was determined using X-tile software (version 3.6.1, Yale University) [25], which identifies the threshold that maximizes the log-rank χ^2^ statistic for survival differences between the high- and low-risk groups. All cut-off selection procedures were performed exclusively on the training set, and no test-set data were accessed or used during any step of the threshold-selection process. The resulting cut-off values derived from the training set were then directly applied to the test set for risk stratification. Survival differences between these stratified groups were visualized using Kaplan–Meier (KM) curves and formally compared using the log-rank test. Furthermore, time-dependent receiver operating characteristic (ROC) analysis was performed to evaluate and compare the predictive accuracy of each model at specific, clinically relevant follow-up intervals (e.g., 1, 2, and 3 years). For the final multi-modal combined model, calibration curves and decision curve analysis (DCA) were additionally utilized to assess its calibration performance and clinical net benefit, respectively.

To explore the potential clinical utility of the combined model, a nomogram was constructed based on the independent prognostic factors identified in the combined model. The nomogram integrated the clinical, radiomic, and DL2D signatures to quantitatively predict the 1-, 2-, and 3-year PFS probabilities for individual DLBCL patients. The points assigned to each variable in the nomogram were determined according to the regression coefficients of the multivariate Cox model, with higher points corresponding to a higher prognostic risk.

### 2.8. Statistical Analysis

The normality of continuous variables was assessed using the Shapiro–Wilk test. Inter-group comparisons were conducted using independent-sample *t*-tests for normally distributed variables or Mann–Whitney U tests for non-normally distributed variables. Categorical variables were compared using the χ^2^ test or Fisher’s exact test, as appropriate. Survival analyses were conducted using Cox proportional hazard regression models.

All statistical analyses were conducted using Python (version 3.7.12). Specifically, the Statsmodels (version 0.13.2) package was used for statistical modeling, Scikit-learn (version 1.0.2) for machine learning pipelines, and PyTorch (version 1.11.0) with CUDA 11.3.1 and cuDNN 8.2.1 acceleration for DL2D model development. Radiomic feature extraction was performed with PyRadiomics (version 3.0.1). The 95% confidence intervals (CI) for C-index values were estimated using bootstrap resampling with 2000 iterations. To compare the prognostic performance between models, we calculated the difference in C-index (ΔC-index) with 95% CI using 2000 bootstrap. The 95% CI were obtained from the 2.5th and 97.5th percentiles of the bootstrap distribution. Time-dependent ROC analysis was performed using Python with standard survival analysis libraries, and censored data were handled using inverse probability of censoring weighting (IPCW). A two-tailed *p* < 0.05 was considered statistically significant.

## 3. Results

### 3.1. Baseline Clinical Characteristics

A total of 281 patients were enrolled in this study and randomly stratified into a training set (*n* = 196, 70%) and a test set (*n* = 85, 30%). Comparison of baseline clinical characteristics revealed no significant differences between the two cohorts in terms of gender, age, extranodal involvement, LDH level, ECOG performance status, B symptoms, bulky disease, cell of origin and Bcl-2/MYC double expression (all *p* > 0.05). A statistically significant between-group difference was only observed in Ann Arbor stage at baseline (*p* = 0.009), which did not compromise the overall baseline balance or the reliability of subsequent model development and analysis. Ann Arbor stage was adjusted as a covariate in the subsequent multivariate analysis to eliminate its confounding effect on the prognostic results. Baseline clinical characteristics of the training and test sets are summarized in Table 1.

### 3.2. Univariable and Multivariable Cox Regression Analysis of Clinical Features

Univariable Cox regression analysis revealed that age (HR (Hazard Ratio) = 1.504, 95% CI: 1.021–2.217, *p* = 0.039), Ann Arbor stage (HR = 1.803, 95% CI: 1.408–2.310, *p* < 0.001), LDH level (HR = 1.635, 95% CI: 1.113–2.403, *p* = 0.012), bulky disease (HR = 2.066, 95% CI: 1.407–3.033, *p* < 0.05), and Bcl-2/MYC double expression (HR = 1.905, 95% CI: 1.300–2.791, *p* < 0.001) were significant prognostic factors for patient outcomes.

In the subsequent multivariable Cox regression model, Ann Arbor stage (HR = 1.650, 95% CI: 1.266–2.150, *p* < 0.001), and bulky disease (HR = 1.656, 95% CI: 1.101–2.491, *p* = 0.015) remained independent adverse prognostic factors. The prognostic significance of Bcl-2/MYC double expression became marginally significant in the multivariable analysis (HR = 1.502, 95% CI: 1.009–2.238, *p =* 0.045) (Table 2).

### 3.3. Baseline Ultrasound Characteristics

Ultrasound features of the 281 enrolled patients were analyzed for the entire cohort and stratified by the training and test sets. No significant between-group differences were observed in long diameter, short diameter, diameter ratio, or other ultrasound characteristics (lymph node location, boundary, shape, hilum of lymph node, reticular echogenicity, blood flow pattern, elastography score, and vascular index; all *p* > 0.05). The baseline ultrasound features of all enrolled patients are presented in Table 3.

### 3.4. Univariable and Multivariable Cox Regression Analysis of Ultrasound Features

Univariable Cox regression analysis of ultrasound features demonstrated that long diameter (HR = 1.023, 95% CI: 1.002–1.044, *p* = 0.034), hilus of lymph node (HR = 1.672, 95% CI: 1.138–2.457, *p* = 0.009), blood flow pattern (HR = 1.613, 95% CI: 1.360–1.912, *p* < 0.001), and vascular index (HR = 1.547, 95% CI: 1.057–2.264, *p* = 0.025) were significant prognostic factors.

In the subsequent multivariable Cox regression model, long diameter (HR = 1.025, 95% CI: 1.005–1.046, *p* = 0.014), hilus of lymph node (HR = 1.794, 95% CI: 1.146–2.809, *p* = 0.011), blood flow pattern (HR = 1.734, 95% CI: 1.409–2.133, *p* < 0.001), and vascular index (HR = 1.629, 95% CI: 1.062–2.509, *p* = 0.026) remained independent adverse prognostic factors (Table 4).

### 3.5. Radiomic Signature Construction and Coefficient Visualization

**Figure 2:**
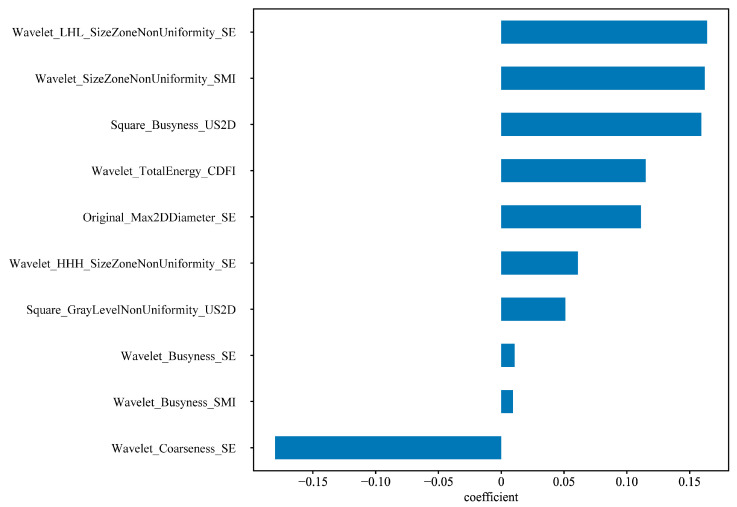
Distribution of weights of radiomic features identified by LASSO-Cox regression with 10-fold cross-validation.

After the three-step hierarchical feature selection, the LASSO-Cox regression model with 10-fold cross-validation identified a subset of radiomic features with non-zero coefficients for the construction of the final radiomic signature. Univariate Cox regression initially identified 32 prognostic radiomic features, including 3 square features, 3 shape features, 1 gradient transform feature, 1 exponential transform feature, and 24 wavelet transform features. The LASSO-Cox regression model selected 10 features to construct the final radiomic signature. The distribution of radiomic feature weights is illustrated in Figure 2. The regression coefficients of these selected features are visualized as a bar chart, which intuitively reflects the direction and magnitude of weights of each feature associated with PFS in patients with DLBCL. Among the selected radiomic features, one feature presented a negative weight, indicating a prognostic protective effect on patient PFS; the remaining features all had positive weights, suggesting an association between these features and an increased risk of disease progression.

### 3.6. Interpretability Analysis of the DL2D Model by Grad-CAM

**Figure 3:**
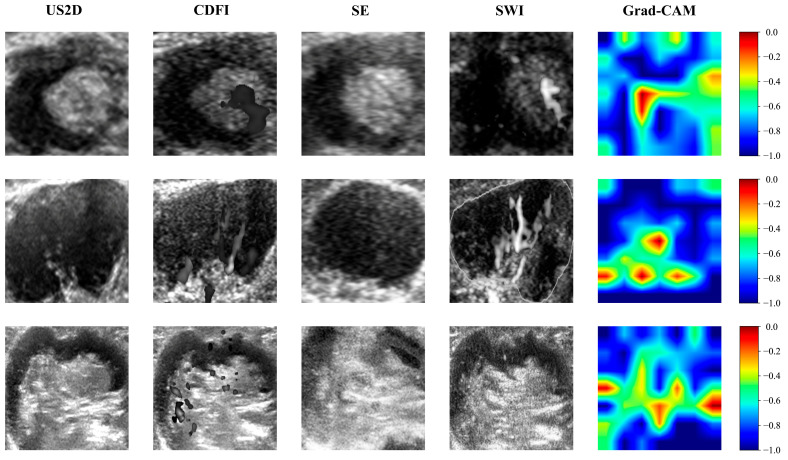
Grad-CAM activation maps of ultrasound images for DLBCL prognostic prediction.

Grad-CAM was applied to visualize the critical ultrasound image regions focused on by the DL2D model for PFS prognostic prediction. Representative activation maps are shown in Figure 3. These maps suggested that the model’s attention was not strictly confined to the manually delineated tumor ROI, but rather appeared to encompass both intratumoral areas (e.g., core parenchyma and heterogeneous texture) and peritumoral regions, indicating that the model may leverage complementary information from the tumor and its surrounding context.

### 3.7. C-Index Comparison of Prognostic Models

In the training cohort, the C-index values for the competing models were as follows: clinical model, 0.714 (95% CI, 0.666–0.762); US model, 0.717 (95% CI, 0.656–0.776); radiomic model, 0.723 (95% CI, 0.665–0.779); DL2D model, 0.796 (95% CI, 0.750–0.839); and the combined model, 0.819 (95% CI, 0.774–0.860). In the test cohort, the C-index values were 0.609 (95% CI, 0.516–0.699) for the clinical model, 0.632 (95% CI, 0.513–0.740) for the US model, 0.757 (95% CI, 0.661–0.845) for the radiomic model, 0.777 (95% CI, 0.705–0.844) for the DL2D model, and 0.811 (95% CI, 0.734–0.876) for the combined model (Table 5). Pairwise comparisons of model performance (ΔC-index with 95% CI) based on 2000 bootstrap are provided in Supplementary Table S1.

### 3.8. Risk Stratification Performance by KM Survival Curves

**Figure 4:**
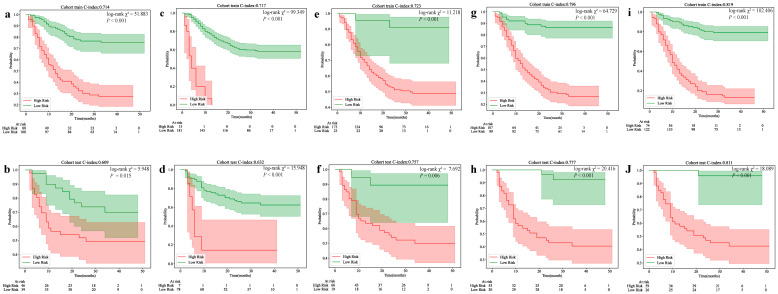
Kaplan–Meier survival curves for different predictive signatures across the training and test cohorts, illustrating risk stratification performance and survival separation between high- and low-risk groups. (**a**,**b**) show the clinical model in the training and test cohorts, respectively, (**c**,**d**) the ultrasound model, (**e**,**f**) the radiomic model, (**g**,**h**) the DL2D model, and (**i**,**j**) the combined model, with red curves for the high-risk group and green curves for the low-risk group.

Based on the optimal cut-off values of risk scores derived from each prognostic model (clinical model: 1.089; ultrasound model: 3.709; radiomic model: 0.397; DL2D model: 0.577; combined model: 1.308; Table 6), patients in both the training and test cohorts were stratified into high- and low-risk groups. KM survival curves were generated to compare PFS between the two groups, and intergroup differences were statistically compared using the log-rank test (Figure 4). All models effectively distinguished between high- and low-risk DLBCL patients, with the high-risk groups exhibiting significantly shorter PFS than the low-risk groups across all models (all *p* < 0.01). Notably, the combined model achieved the most robust risk stratification performance, showing the most distinct separation of KM curves between high- and low-risk groups in both cohorts (log-rank χ^2^ = 18.089, *p* < 0.001 for the test cohort).

### 3.9. Time-Dependent ROC Analysis for PFS Prediction

**Figure 5:**
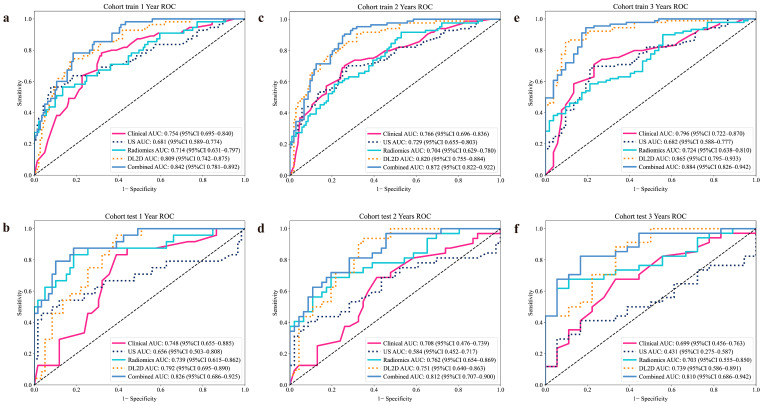
Time-dependent ROC curves for PFS prediction in the training and test cohorts at different time points. (**a**,**b**) show 1-year PFS prediction in the training and test cohorts, respectively; panels (**c**,**d**) show 2-year PFS prediction; (**e**,**f**) show 3-year PFS prediction. Curves represent the clinical model, US model, radiomics model, DL2D model, and combined model, with the AUC and 95% CI reported for each model.

ROC curve analysis was performed to evaluate the predictive performance of clinical, US, radiomic, DL2D, and combined models for 1-, 2-, and 3-year PFS, and the results showed that the combined model consistently achieved the highest AUC values at all time points in both the training and test cohorts, with AUCs of 0.842 (95% CI: 0.781–0.892) for 1-year PFS, 0.872 (95% CI: 0.822–0.922) for 2-year PFS, and 0.884 (95% CI: 0.826–0.942) for 3-year PFS in the training cohort, and 0.826 (95% CI: 0.686–0.925) for 1-year PFS, 0.812 (95% CI: 0.707–0.900) for 2-year PFS, and 0.810 (95% CI: 0.686–0.942) for 3-year PFS in the test cohort, all significantly higher than single-modality models (Figure 5).

### 3.10. DCA of Prognostic Models

**Figure 6:**
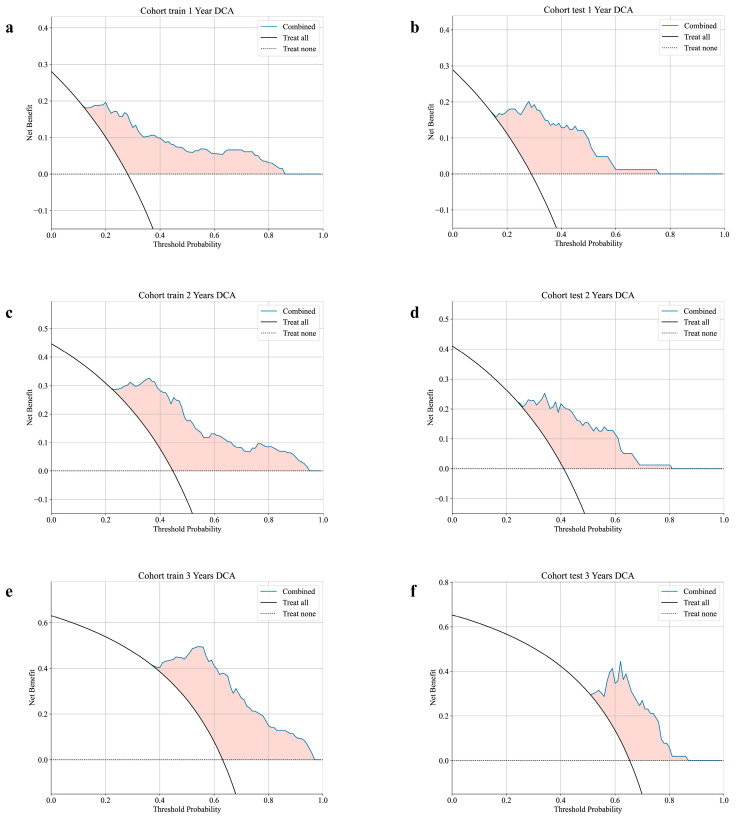
DCA of the combined model for 1-, 2-, and 3-year PFS prediction. (**a**,**b**) show 1-year PFS prediction in the training and test cohorts, respectively; (**c**,**d**) show 2-year PFS prediction; (**e**,**f**) show 3-year PFS prediction.

DCA was performed to evaluate the clinical net benefit of the multi-modal combined model for predicting 1-, 2-, and 3-year PFS (Figure 6). Across all time points in both cohorts, the combined model achieved a higher net benefit than the extreme clinical strategies of “treating all patients” and “treating no patients” within the clinically relevant threshold range (approximately 0.2–0.8). In the low-threshold range, the “treat all” strategy temporarily showed higher net benefit, but the combined model consistently outperformed both extreme strategies across the threshold range most relevant to clinical practice. The combined model demonstrated acceptable calibration for 1-, 2-, and 3-year PFS predictions.

### 3.11. Development and Validation of a Nomogram for PFS Prediction

**Figure 7:**
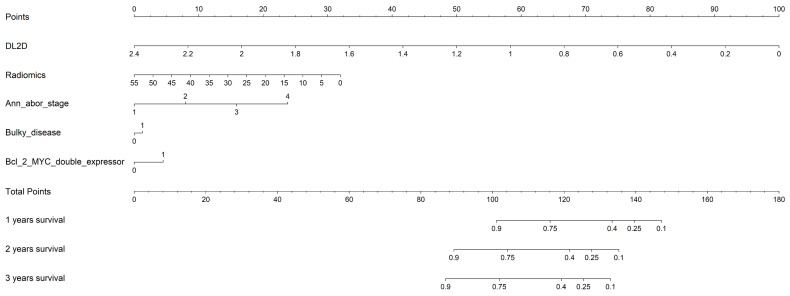
Nomogram for predicting 1-, 2-, and 3-year PFS in patients with DLBCL.

A clinical nomogram was constructed based on the combined model (Figure 7) to explore the feasibility of visually and quantitatively estimating the 1-, 2-, and 3-year PFS of DLBCL patients. This nomogram integrated independent prognostic covariates, including the clinical signature, radiomic signature, and DL2D signature. Each variable was assigned a specific point value on the nomogram scale according to its regression coefficient in the multivariate Cox model, and the total points of an individual patient were calculated by summing the points of all covariates. The total points were then mapped to the corresponding scale to obtain the estimated 1-, 2-, and 3-year PFS probabilities.

## 4. Discussion

Unlike PET/CT-based models, our approach used widely available, low-cost, radiation-free ultrasound, making it particularly suitable for resource-limited settings and primary hospitals. Ultrasound could provide excellent high-resolution imaging of superficial soft tissues and allow for clear visualization of lymph node morphology, Doppler flow, and other indicators. It thereby enables AI to reliably extract high-throughput quantitative features that cannot be fully captured by conventional subjective assessment. Previous studies have also confirmed that ultrasound AI features of superficial lymph nodes closely reflect tumor biological behavior, treatment response, and long-term survival [16,17,26], providing a reliable theoretical basis for constructing ultrasound-based prognostic models in this study.

DLBCL exhibits substantial prognostic heterogeneity, and accurate risk stratification is critical for optimizing individualized treatment. With the continuous advancement of precision medicine, traditional prognostic models based on single-dimensional information can no longer meet clinical requirements; so, the construction of predictive models integrating multi-modal information has become a research hotspot in recent years [27,28]. Based on the characteristic that most patients with DLBCL present with involvement of superficial lymph nodes in the cervical, axillary, and inguinal regions as the initial or main clinical manifestation [23], this study selected patients with superficial lymph node involvement in these regions as the targets for imaging feature mining.

To address this clinical demand, the present study integrated ultrasound radiomic features, deep learning features, and clinical indicators to construct a combined multi-feature prognostic model, and further developed a corresponding nomogram for predicting 1-, 2-, and 3-year PFS in patients with DLBCL. Our results demonstrated that the C-index of the combined model reached 0.811, and its predictive performance was significantly superior to that of the clinical-only model and conventional ultrasound-related models, enabling effective PFS assessment. Meanwhile, using the risk score derived from this model, patients with DLBCL could be stratified into high- and low-risk groups. The nomogram may assist in prognostic evaluation and provide a quantitative reference for risk stratification.

### 4.1. Radiomic Model

Traditional clinical prognostic models, such as IPI, mainly rely on clinical indicators to assess the prognosis of patients, but ignore the morphological and biological characteristics of the tumor itself, resulting in limited predictive efficiency [29,30]. In this study, the C-index of the clinical-only model was 0.609, which was consistent with the predictive efficiency of IPI and its derived scoring systems in previous studies. Although conventional ultrasound can provide basic image information such as lesion morphology, elasticity and blood flow, it is mostly qualitative description with strong subjectivity and insufficient information mining, and the C-index of its model was only 0.632. It failed to significantly improve the predictive efficiency, which further showed the limitations of traditional imaging in capturing prognostic information [19,20]. The C-index of the ultrasound radiomic model in this study was 0.757, higher than that of the conventional ultrasound model, indicating that radiomic features were able to capture more prognosis-related information, such as the uniformity of tumor gray distribution and texture complexity.

It was demonstrated that these features were closely related to tumor invasiveness and proliferative capacity [12,13,19]. Another study has confirmed that ultrasound radiomic features could indirectly reflect the proliferative activity and angiogenesis status of tumor cells by quantifying the heterogeneity of the tumor microenvironment [11]. The 32 radiomic features identified by univariate Cox regression in this study—including 3 square features, 3 shape features, 1 gradient transform feature, 1 exponential transform feature, and 24 wavelet transform features—were consistent with the biological significance underlying tumor heterogeneity. Specifically, the shape features encompassing maximum diameter and sphericity objectively assess the tumor growth pattern and local infiltration scope, which are positively correlated with the risk of disease progression in patients with DLBCL. The wavelet transforms feature, square feature, gradient transform feature and exponential transform feature, as the core quantitative components, effectively quantifies the subtle heterogeneity of intratumoral gray distribution, and further reflects the disordered arrangement of tumor cells and the corresponding invasive potency, which supports the reliability of the extracted radiomic features in reflecting tumor biological behavior [31].

Subsequently, multivariate LASSO-Cox regression further reduced these to 10 core features, which were used to evaluate PFS and predict the risks of recurrence, progression and death in DLBCL patients. Most of the screened features were wavelet transform features, which exhibited the highest weight coefficients in the prognostic model. These features excelled at capturing multi-scale spatial information and subtle intratumoral heterogeneity in ultrasound images. They strongly indicated that high-frequency details within ultrasound images played a core role in the prognostic evaluation of DLBCL. This finding further supports the biological interpretability of the radiomic features selected in this study [19,32,33].

### 4.2. DL2D Model

Traditional radiomic methods have shown potential in many aspects, but there are still limitations in the automation, standardization and efficiency of feature extraction, especially in capturing advanced nonlinear features of images [34]. To make up for these deficiencies, comprehensively mining the prognosis-related information contained in tumor images probably improves the predictive efficiency of the model. Our results showed a C-index of 0.777 for the deep learning model, which was significantly better than that of the traditional radiomic model. This finding was in agreement with the advantages of deep learning in tumor image-based prognosis prediction reported in previous studies [35,36].

In this study, a transfer learning strategy was adopted for deep feature extraction, and the pre-trained robust deep neural network model DenseNet121 was selected. With its residual connection structure, this model could effectively solve the gradient vanishing problem in the training of deep networks and has stronger feature representation ability. The studies have shown that the DenseNet121 model has exhibited excellent performance in the imaging analysis of thyroid cancer, breast cancer and other tumors [37,38,39]. In this study, the deep learning model constructed based on four modal ultrasound images (US2D, CDFI, SMI and SE) had significant predictive advantages in various evaluation indicators compared with the traditional radiomic model, with C-index values increased by 0.054. This highlighted the advantages of deep learning in mining prognostic information from DLBCL patients’ images and further improved the accuracy of the predictive model.

Notably, to improve the interpretability of our DL2D model and address the “black box” limitation common in DL2D-based imaging prognostic models, we applied Grad-CAM to visualize the key regions of ultrasound images that the DenseNet121 model focused on for PFS prediction [40]. The results suggested that our model appeared to focus on both intratumoral regions and peritumoral areas, indicating that it may leverage complementary information from the tumor and its surrounding context for prognostic prediction.

The C-index of the DL2D model in this study reached 0.777, higher than that reported in the multicenter study by Jiang et al. [41]. However, considering that this study was a single-center design and the clinical accessibility of the ultrasound modality was stronger, this result suggested the clinical advantages of the deep learning model in capturing prognostic information. This was consistent with the viewpoint proposed by Cheng et al. [42] regarding the potential clinical utility of ultrasound combined with AI technology, and made up for the deficiency of current ultrasound DL2D prognostic studies on DLBCL. To enhance the model’s robustness, generalization ability and adaptability to DLBCL-specific imaging features, we adopted a comprehensive optimization strategy based on transfer learning for DenseNet121: we performed end-to-end fine-tuning of all network layers and adopted a dynamically and precisely adjusted learning rate strategy. This optimization not only allowed the network to efficiently extract high-dimensional, complex deep features characterizing tumor pathological heterogeneity, but also targeted the overfitting issue induced by the limited sample size in this single-center study. Consistent with the optimization scheme for small-sample imaging AI models proposed by Yao et al. [43] and referencing the multicenter ultrasound deep learning method by Qi et al., this integrated strategy enhanced the model’s robustness and the reliability of internal validation [44].

### 4.3. Multi-Modal Ultrasound Imaging

Among the ten core features, SE contributed the most with five features, highlighting its irreplaceable value in reflecting the mechanical properties of tumor tissues. SE-derived features, especially those capturing tissue stiffness heterogeneity, are closely correlated with critical pathological processes in DLBCL, such as tumor cell proliferation, interstitial fibrosis, and tissue invasiveness [21]. The abundance of effective features from SE confirmed that mechanical characteristics were key prognostic indicators for DLBCL, providing a novel perspective beyond traditional morphological evaluation.

Following SE, US2D and SMI each contributed two features, forming a complementary partnership with SE. US2D provided fundamental morphological information, while SMI complements CDFI by visualizing microvascular distribution more sensitively. Notably, CDFI only contributed one feature (total energy), which may be attributed to the unstable or inconsistent vascular distribution in most DLBCL lesions, limiting the extraction of stable and quantitative features.

The integration of US2D, CDFI, SE, and SMI constituted a comprehensive multi-modal ultrasound radiomic strategy. This multi-modal approach overcame the limitations of single-modal imaging by simultaneously capturing morphological, mechanical, and microvascular pathological characteristics of tumors, thereby constructing a more holistic and accurate prognostic signature. This multi-modal fusion strategy represented the key innovation of this study, as it achieved a comprehensive and in-depth characterization of DLBCL lesions, potentially improving the prognostic utility of the PFS prediction model. A previous breast cancer radiomic study also found that SE-based ultrasound models outperformed other single-modal models, highlighting the clinical value of this imaging modality for tumor survival prediction [45].

Building on this multi-modal ultrasound radiomic strategy, we further explored the application of multi-modal fusion in deep-learning-based prognostic modeling, and achieved a more in-depth excavation of lesion prognostic information. For the DL2D-based prognostic modeling in this study, we further leveraged the complementary advantages of the four-modal ultrasound images (US2D, CDFI, SMI, and SE) by constructing a dedicated multichannel input structure. Specifically, axial image slices of each modal sequence were cropped based on tumor ROIs and stacked to form a four-channel composite input tensor, which enabled the DenseNet121-based DL2D model to synchronously learn and integrate morphological, hemodynamic, microvascular, and mechanical feature information of DLBCL lesions in a unified network representation. Unlike the manual feature engineering in radiomics, the DL2D model autonomously mines high-dimensional, nonlinear, and cross-modal associated with deep features from the fused multi-modal ultrasound data—features that are difficult to identify and extract through traditional radiomic methods. This end-to-end multi-modal feature learning mode not only avoids the information loss caused by artificial feature screening but also fully excavates the potential prognostic information hidden in the interaction of different ultrasound modal features, thus further enhancing the model’s ability to characterize tumor pathological heterogeneity and predict PFS [39].

This multi-modal fusion-based deep learning strategy also echoes the research advances in medical imaging AI, where the integration of multi-modal data has been proven to effectively improve the predictive performance of DL2D models for various tumors [23]. By combining the strengths of multi-modal ultrasound and deep learning, our study realized a more comprehensive and in-depth mining of DLBCL lesion information than single-modal deep learning models or traditional multi-modal radiomics, which constitutes another important innovation of our research following the multi-modal radiomic strategy. It further confirmed that the combination of multi-modal ultrasound imaging and deep learning was a promising approach for prognostic prediction in DLBCL, and provided a potential approach for prognostic risk assessment in DLBCL patients.

### 4.4. Combined Model

The core highlight of the combined model constructed in this study was the integration of three dimensions: clinical indicators, ultrasound radiomic features and deep learning features, realizing the complementary fusion of multi-modal information. Traditional radiomic methods extract first-order statistical and second-order texture features based on lesion gray histograms, which quantify the basic heterogeneity of tumors; deep learning networks mine high-dimensional and nonlinear deep features from tumor regions, capturing the hidden complex correlational information in images; clinical indicators reflect the overall health status and disease burden of patients. The synergistic effect of the three significantly improved the predictive efficiency of the model. The C-index of the combined model reached 0.811, with AUC values of 0.826, 0.812 and 0.810 for the prediction of 1-year, 2-year and 3-year PFS, respectively, and a good risk stratification effect between the high-risk and low-risk groups. This was consistent with the research conclusions of Lu et al. [19] and Zhao et al. [46], confirming the core value of multi-modal ultrasound images in tumor prognosis prediction. Notably, this study optimized the LASSO-Cox screening parameters using 10-fold cross-validation, which significantly reduced the random error of feature selection compared with simple random splitting, thus ensuring the stability and repeatability of key features [47].

Compared with similar combined models, most of the current multi-modal prognostic studies on DLBCL focused on the combination of PET-CT and clinical features. For example, the model by Wang et al. [6] integrating PET-CT deep learning features and multi-omics data had a C-index of 0.70, while the 1-year, 2-year and 3-year AUC of the combined model in this study reached more than 0.80 with better predictive efficiency. DCA further validated the prognostic utility of the combined model, which exhibited a significant positive clinical net benefit for PFS prediction in DLBCL patients within the threshold probability interval of 0.2 to 0.8. These results indicated that integrating multi-modal ultrasound-derived tumor information and clinical indicators to construct a combined model could effectively improve the accuracy of prognostic prediction, which was consistent with the research norm proposed by Yang et al. [27] that multi-modal fusion was the key to improving the prognostic performance of models.

On this basis, the nomogram developed in this study serves as an intuitive visualization tool that may facilitate individualized risk estimation. This is consistent with the finding of Jelicic et al. [48] that the ultrasound-based nomogram could serve as a reference for risk assessment in future clinical workflows, and highly consistent with the viewpoint proposed by Li et al. [17] that prognostic models need to develop intuitive visualization tools such as nomograms. Nomograms derived from ultrasound and clinical data have been successfully developed for nodal involvement prediction in other malignancies, such as endometrial cancer [49]. This provides a methodological precedent for constructing an ultrasound-based nomogram for DLBCL PFS prediction in our study. In addition, the combined model in this study was constructed based on the ultrasound modality, with the advantages of non-invasiveness, low cost, no radiation and repeatable examination. In primary hospitals and resource-limited areas, the accessibility of ultrasound examination is significantly higher than that of PET-CT and MRI, which may make the model and nomogram constructed in this study more accessible in resource-limited settings, suggesting a promising avenue for future applications if validated in broader populations [19].

In addition, the selection of 1-to-3-year PFS as the predictive endpoint in this study has sufficient evidence-based basis. Maurer et al. [50] confirmed, based on a large sample DLBCL cohort, that 24-month PFS could be used as a reliable surrogate endpoint for overall survival (OS). It provided strong support for the rationality of endpoint selection in this study. This study focused on the prediction of 1-to-3-year PFS, which not only met the clinical demand for short-term prognostic assessment but could also indirectly reflect long-term survival benefits, avoiding the problem of insufficient clinical practicability caused by too long a follow-up period. This was consistent with the research idea proposed by Cheng et al. that selecting a reasonable prognostic endpoint was the key to improving the clinical application value of the model [42].

### 4.5. Study Limitations and Future Directions

Despite several strengths, this study has some limitations. First, this was a single-center retrospective study with a relatively small sample size, which may lead to selection bias. Therefore, future multicenter, large-sample prospective studies are needed to validate the model’s generalization ability. Additionally, the limited number of Ann Arbor stage I–II cases (*n* = 6) in the test set precluded robust subgroup validation across Ann Arbor stage categories; external validation in larger, more balanced cohorts is warranted. Second, several data-quality limitations should be considered. Ultrasound images were stored in JPG format, a lossy compression that may affect gray-level intensity and texture-based radiomic features. Additionally, multiple ultrasound devices were used without formal harmonization, such as ComBat, and the sample sizes across devices were imbalanced, which limits cross-device generalizability. Future studies using DICOM storage and external, more balanced multi-device validation are warranted. Thus, these findings should be considered preliminary and require further external validation. Third, we included patients with superficial lymph node involvement as the initial or main disease sites (with or without deep nodal/extranodal involvement). As this is the most common presentation of DLBCL, our results are clinically representative. Future studies may combine superficial and deep lymph node and extranodal organ data to expand the model’s applicability. Forth, direct comparison of prognostic performance between ultrasound and PET was not feasible in our retrospective cohort, which may limit the validation of our ultrasound-based model. Therefore, future studies with paired imaging data are warranted. Finally, comparisons with molecular models as well as correlations between imaging biomarkers and biological parameters were not fully analyzed, because complete molecular data were unavailable. Future studies will explore these aspects to further refine the model.

In summary, the combined model integrating ultrasound radiomics, ultrasound deep learning features and clinical indicators showed good predictive value for PFS in DLBCL patients, and its performance was significantly better than that of the other single models, with a C-index of 0.811 and AUC values exceeding 0.80 for 1-, 2-, and 3-year PFS. While these findings are based on a single-center retrospective cohort, the model and nomogram showed promising potential for practical and intuitive prognostic assessment and risk stratification in DLBCL.

## Figures and Tables

**Table 1:** Baseline clinical characteristics of the study cohort and comparison between training and test subsets.

	All	Training	Test	*p* Value
Gender				0.217
Female	138 (49.11%)	91 (46.43%)	47 (55.29%)	
Male	143 (50.89%)	105 (53.57%)	38 (44.71%)	
Age				0.153
≤60 years	129 (45.91%)	84 (42.86%)	45 (52.94%)	
>60 years	152 (54.09%)	112 (57.14%)	40 (47.06%)	
Ann Arbor stage				0.009
I–II	46 (16.37%)	40 (20.41%)	6 (7.06%)	
III–IV	235 (83.63%)	156 (79.59%)	79 (92.94%)	
Extranodal involvement				0.386
No	146 (51.96%)	98 (50.00%)	48 (56.47%)	
Yes	135 (48.04%)	98 (50.00%)	37 (43.53%)	
LDH				0.802
Normal	134 (47.69%)	92 (46.94%)	42 (49.41%)	
Elevated	147 (52.31%)	104 (53.06%)	43 (50.59%)	
ECOG_PS				0.257
0–1	145 (51.60%)	106 (54.08%)	39 (45.88%)	
≥2	136 (48.40%)	90 (45.92%)	46 (54.12%)	
B symptoms				0.217
No	138 (49.11%)	91 (46.43%)	47 (55.29%)	
Yes	143 (50.89%)	105 (53.57%)	38 (44.71%)	
Bulky disease				0.073
No	179 (63.70%)	132 (67.35%)	47 (55.29%)	
Yes	102 (36.30%)	64 (32.65%)	38 (44.71%)	
Cell of origin				0.724
GCB	136 (48.40%)	93 (47.45%)	43 (50.59%)	
Non-GCB	145 (51.60%)	103 (52.55%)	42 (49.41%)	
Bcl-2/MYC double expressor				0.231
No	189 (67.26%)	127 (64.80%)	62 (72.94%)	
Yes	92 (32.74%)	69 (35.20%)	23 (27.06%)	

**Table 2:** Univariable and multivariable analysis of clinical features.

	Univariable Analysis				Multivariable Analysis			
	HR	Lower 95% CI	Upper 95% CI	*p* Value	HR	Lower 95% CI	Upper 95% CI	*p* Value
Gender	1.153	0.791	1.682	0.459				
Age	1.504	1.021	2.217	0.039	1.287	0.861	1.924	0.218
Ann Arbor stage	1.803	1.408	2.310	<0.001	1.650	1.266	2.150	<0.001
Extranodal involvement	0.955	0.656	1.392	0.812				
LDH	1.635	1.113	2.403	0.012	1.398	0.933	2.096	0.105
ECOG PS	1.325	0.909	1.931	0.144				
B symptoms	0.876	0.601	1.276	0.490				
Bulky disease	2.066	1.407	3.033	<0.001	1.656	1.101	2.491	0.015
Cell of origin	1.199	0.822	1.748	0.346				
Bcl2/MYC double expressor	1.905	1.300	2.791	<0.001	1.502	1.009	2.238	0.045

**Table 3:** Baseline ultrasound characteristics of the study cohort and comparison between training and test subsets.

	All	Training	Test	*p* Value
Long diameter (mm)	25.99 ± 9.79	26.09 ± 8.79	25.94 ± 10.22	0.419
Short diameter (mm)	14.43 ± 8.18	14.04 ± 7.13	14.60 ± 8.61	0.634
Ratio	1.87 ± 0.70	1.82 ± 0.68	1.89 ± 0.71	0.332
Location				0.726
Cervical	162 (57.65%)	46 (54.12%)	116 (59.18%)	
Axillary	59 (21.00%)	19 (22.35%)	40 (20.41%)	
Inguinal	60 (21.35%)	20 (23.53%)	40 (20.41%)	
Boundary				>0.999
Clear	196 (69.75%)	59 (69.41%)	137 (69.90%)	
Ill-defined	85 (30.25%)	26 (30.59%)	59 (30.10%)	
Shape				0.252
Oval	176 (62.63%)	58 (68.24%)	118 (60.20%)	
Irregular	105 (37.37%)	27 (31.76%)	78 (39.80%)	
Hilus of lymph node				0.503
Present	132 (46.98%)	43 (50.59%)	89 (45.41%)	
Absent	149 (53.02%)	42 (49.41%)	107 (54.59%)	
Reticular echogenicity				0.562
Absent	153 (54.45%)	49 (57.65%)	104 (53.06%)	
Present	128 (45.55%)	36 (42.35%)	92 (46.94%)	
Blood flow pattern				0.916
No flow	50 (17.79%)	17 (20.00%)	33 (16.84%)	
Central (hilar) flow	55 (19.57%)	15 (17.65%)	40 (20.41%)	
Mixed flow	72 (25.62%)	23 (27.06%)	49 (25.00%)	
Peripheral flow	76 (27.05%)	21 (24.71%)	55 (28.06%)	
Disordered flow	28 (9.96%)	9 (10.59%)	19 (9.69%)	
Elastography score				0.442
1	13 (4.63%)	4 (4.71%)	9 (4.59%)	
2	160 (56.94%)	54 (63.53%)	106 (54.08%)	
3	92 (32.74%)	22 (25.88%)	70 (35.71%)	
4	16 (5.69%)	5 (5.88%)	11 (5.61%)	
Vascular index				0.670
Low	145 (51.60%)	46 (54.12%)	99 (50.51%)	
High	136 (48.40%)	39 (45.88%)	97 (49.49%)	

**Table 4:** Univariable and multivariable Cox regression analysis of ultrasound features.

	Univariable Analysis				Multivariable Analysis			
	HR	Lower 95% CI	Upper 95% CI	*p* Value	HR	Lower 95% CI	Upper 95% CI	*p* Value
Long diameter	1.023	1.002	1.044	0.034	1.025	1.005	1.046	0.014
Short diameter	1.013	0.993	1.034	0.201				
Ratio	0.973	0.741	1.280	0.847				
Location	0.978	0.775	1.234	0.853				
Boundary	1.028	0.680	1.554	0.896				
Shape	0.913	0.620	1.346	0.647				
Hilus of lymph node	1.672	1.138	2.457	0.009	1.794	1.146	2.809	0.011
Reticular echogenicity	1.049	0.719	1.530	0.803				
Blood flow pattern	1.613	1.360	1.912	<0.001	1.734	1.409	2.133	<0.001
Elastography score	1.122	0.843	1.494	0.428				
Vascular index	1.547	1.057	2.264	0.025	1.629	1.062	2.509	0.026

**Table 5:** Comparison of C-index values between clinical, ultrasound, radiomic, DL2D, and combined models.

Cohort	Clinical Model	Ultrasound Model	Radiomic Model	DL2D Model	Combined Model
Training	0.714 (0.666–0.762)	0.717 (0.656–0.776)	0.723 (0.665–0.779)	0.796 (0.750–0.839)	0.819 (0.774–0.860)
Test	0.609 (0.516–0.699)	0.632 (0.513–0.740)	0.757 (0.661–0.845)	0.777 (0.705–0.844)	0.811 (0.734–0.876)

**Table 6:** Optimal thresholds for risk stratification (KM analysis).

Cohort	Clinical Model	Ultrasound Model	Radiomic Model	DL2D Model	Combined Model
Training	1.089	3.709	0.397	0.577	1.308

## Data Availability

The original contributions presented in this study are included in this article. Further inquiries can be directed to the corresponding author.

## Supplementary Materials

The following supporting information can be downloaded at: https://www.mdpi.com/article/10.3390/diagnostics16152311/s1, Supplementary Table S1: Comparison of prognostic performance between models using ΔC-index with 95% CI based on 2000 bootstrap. Supplementary Figure S1: Training and test loss curves of the DL2D model.
