# Supplementary material for: Multi-Modal Ultrasound-Based Prognostic Model for Diffuse Large B-Cell Lymphoma with Predominantly Superficial Lymph Node Involvement
Source: Diagnostics (Basel). 2026 Jul 23;16(15):2311. doi: 10.3390/diagnostics16152311 (PMC13465404; doi:10.3390/diagnostics16152311)
Supplement: Supplementary file 1 [file diagnostics-16-02311-s001.zip › Supplement Table S1.pdf]

Supplementary Table S1. Comparison of prognostic performance between models using ΔC-index with 95% confidence intervals based on 2000 bootstrap

**Cohort**	**Radiomics vs. Clinical**	P	**DL2D vs. Clinical**	P	**Combine vs. Clinical**	P
Training	0.008 (-0.061-0.074)	0.811	0.082(0.023-0.142)	0.007	0.105 (0.053-0.158)	<0.001
Test	0.148 (0.007-0.283)	0.034	0.168(0.047-0.282)	0.006	0.202 (0.086-0.318)	<0.001

*ΔC-index represents the improvement in C-index relative to the Clinical model. Positive values indicate better predictive performance. *P* values were calculated using the bootstrap method. CI, confidence interval; DL2D, two-dimensional deep learning.
